# Urinary Calculus in a Child

**Published:** 1834

**Authors:** 


### XI.—Large Urinary Calculus in a young Female.

Two or three months ago, a little girl 11 years old, was brought to us to be treated for the "gravel"—considerable quantities of which, it was said, she had been discharging for some years. She could not retain her urine, which escaped as fast as secreted; suffered great pain in the hypogastric and lumbar regions; and passed such afflicting nights, as to be obliged to take large portions of laudanum. This of course produced habitual constipation, but otherwise her health was better than might have been expected, under such acute sufferings, which had continued for about *seven* years.

On examination, we found a calculus projecting out of the external orifice of the urethra, and it was easy to perceive, that it filled the bladder. By the aid of a small pair of lithotomy forceps, it was broken up, and extracted in fragments, the last of which adhered to the fundus of the bladder. As the orifice of the urethra was greatly and permanently dilated by the pressure of the calculus, it was not difficult to introduce the finger, and ascertain that all the fragments were removed. The bladder was then syringed out with tepid water, large quantities of which were thrown in, and an opiate administered. No inflammation supervened, and the little patient declared, that the ensuing night, was the most comfortable she had experienced for years. The object of our notice is not, however, to set forth these facts, but the following:

On examining the calculus, it was found to be composed of *uric acid*. The parents of this child had resided from her infancy, on the banks of the Great Sandy, a river of the eastern part of Kentucky, which flows through a secondary sand stone formation, and the water, she had always drunk, was of course soft.

